# Cross sectional evaluation of the gut-microbiome metabolome axis in an Italian cohort of IBD patients

**DOI:** 10.1038/s41598-017-10034-5

**Published:** 2017-08-25

**Authors:** Maria Laura Santoru, Cristina Piras, Antonio Murgia, Vanessa Palmas, Tania Camboni, Sonia Liggi, Ivan Ibba, Maria Antonia Lai, Sandro Orrù, Sylvain Blois, Anna Lisa Loizedda, Julian Leether Griffin, Paolo Usai, Pierluigi Caboni, Luigi Atzori, Aldo Manzin

**Affiliations:** 1 Department of Biomedical Sciences, University of Cagliari, Cagliari, Italy; 2 Department of Life and Environmental Sciences, University of Cagliari, Cagliari, Italy; 3 Department of Medical Sciences and Public Health, University of Cagliari and Gastroenterology Unit, University Hospital of Cagliari, Cagliari, Italy; 4 Gastroenterology Unit, University Hospital of Cagliari, Cagliari, Italy; 5 Consiglio Nazionale delle Ricerche (C.N.R.), Istituto di Ricerca Genetica e Biomedica (I.R.G.B.), Monserrato, Cagliari, Italy; 6 Department of Biochemistry, University of Cambridge, Cambridge, UK

## Abstract

Inflammatory bowel disease (IBD) is a chronic inflammatory disease of the gastrointestinal tract of uncertain origin, which includes ulcerative colitis (UC) and Crohn’s disease (CD). The composition of gut microbiota may change in IBD affected individuals, but whether dysbiosis is the cause or the consequence of inflammatory processes in the intestinal tissue is still unclear. Here, the composition of the microbiota and the metabolites in stool of 183 subjects (82 UC, 50 CD, and 51 healthy controls) were determined. The metabolites content and the microbiological profiles were significantly different between IBD and healthy subjects. In the IBD group, Firmicutes, Proteobacteria, Verrucomicrobia, and Fusobacteria were significantly increased, whereas Bacteroidetes and Cyanobacteria were decreased. At genus level *Escherichia*, *Faecalibacterium*, *Streptococcus*, *Sutterella* and *Veillonella* were increased, whereas *Bacteroides*, *Flavobacterium*, and *Oscillospira* decreased. Various metabolites including biogenic amines, amino acids, lipids, were significantly increased in IBD, while others, such as two B group vitamins, were decreased in IBD compared to healthy subjects. This study underlines the potential role of an inter-omics approach in understanding the metabolic pathways involved in IBD. The combined evaluation of metabolites and fecal microbiome can be useful to discriminate between healthy subjects and patients with IBD.

## Introduction

Inflammatory bowel disease (IBD) represents a group of chronic disorders that affect one or more parts of the intestine. Crohn’s disease (CD) and ulcerative colitis (UC) are considered the two major clinically defined forms^1^. IBD affects 1.5 million Americans, 2.2 million European, and several hundred thousand elsewhere in the world^2^. The highest incidences of CD and UC have been reported in northern Europe, the United Kingdom, and North America with a greater incidence of UC than CD^3^. However, the incidence of IBD is increasing in emerging populations such as Asian, probably due to changes in environmental factors. The peak incidence of IBD is between the second to fourth decades of life^4^. Incidence in established populations is similar in men and women, but is influenced by race and ethnicity.

The etiology of IBD is multifactorial and only partially known, resulting from genetic, immunological and environmental factors. The most accepted hypothesis for the pathogenesis of IBD is the development of an over aggressive adaptive immune response mediated by T cells to a subset of commensal enteric bacteria in genetically susceptible hosts^5^. Moreover, environmental factors, such as diet, life style and smoking play crucial roles in the development of disease^6^. In particular, the relationship between the metabolome and microbiota, such the association between butyrate and Ruminococcus and Butyricicoccus bacteria, has been shown to be of fundamental importance in elucidating IBD pathogenesis and in establishing targeted therapeutic strategies^7,8^. Twin-pair studies have shown differences in fecal metabolites and microbiome between patients with Crohn disease and their healthy siblings^9,10^. Moreover, the detection of metagenomics differences yielded the identification of disease-related biomarkers, which presumably derive from disease-associated alterations in the microbial flora^11^.

In this study, we have used a combined metabolomics and metagenomics approach to study the differences between CD and UC, and understand how these two pathologies arise. Fecal samples of IBD patients, with either CD and UC, and healthy subjects were analyzed by the means of metabolomics and metagenomics. Nuclear magnetic resonance spectroscopy (^1^H-NMR), gas chromatography mass spectrometry (GC-MS) and liquid chromatography in combination with quadrupole time-of-flight mass spectrometry (LC-QTOF-MS) were used to construct the metabolic profile of fecal samples, and 16 S rRNA gene sequencing data were produced from each biospecimen by the Illumina Hi-Seq platform to reveal the gut microflora composition. Finally, we investigated the correlation between measured metabolites and identified bacteria to better define the gut-microbiome metabolome during the development of IBD.

## Results

### Microbiota analysis

**Figure 1:**
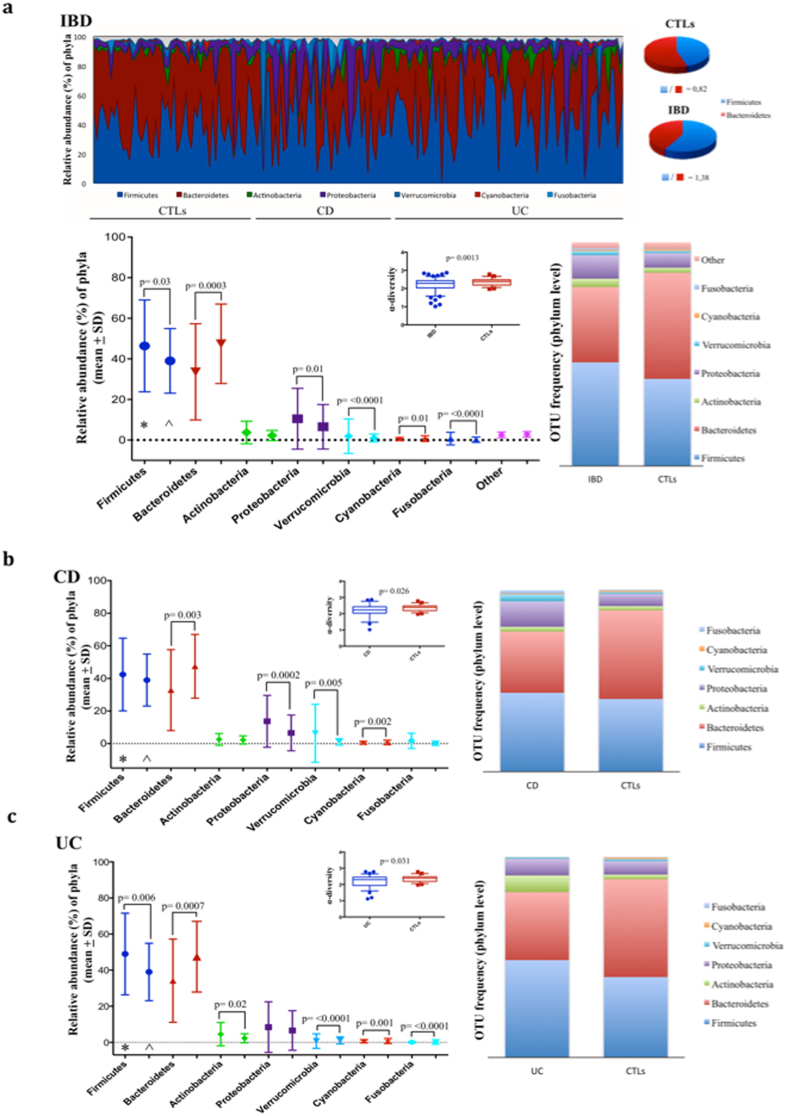
Microbiome taxonomic composition in IBD, CD, UC patients and control subjects (CTLs). Relative abundance at OTU frequency at phylum level, Firmicutes/Bacteroidetes ratio, and α-diversity are shown. The data are filtered by a frequency higher than 0.1% (**a**). Relative abundance of phyla and OTU frequency are shown in CD (*) (**b**) and UC (*) (**c**) patients compared to controls (CTLs, ^). Significant differences with p < 0.05 are shown. *patients; ^controls.

A total of 18,899,323 sequencing reads were obtained from the 183 fecal samples. The numbers of OTUs varied from 4,901 to 672,146. The mean differences in the α-Shannon values between the CTLs, the IBD, and the CD and UC groups were always statistically different (Fig. 1). The majority of the OTU sequences were assigned to seven main phyla: Firmicutes, Bacteroidetes, Actinobacteria, Proteobacteria, Verrucomicrobia, Cyanobacteria and Fusobacteria. In Fig. 1 the relative abundance and frequency of the OTUs at the phylum level are shown for those OTUs that have at least 0.1% abundance. In the IBD group compared to the CTLs group, Firmicutes (46.39% *vs* 38.99%), Proteobacteria (10.49% *vs* 6.54%), Verrucomicrobia (1.90% *vs* 1.02%), and Fusobacteria (0.72% *vs* 0.19%) were significantly increased, whereas Bacteroidetes (33.63% *vs* 47.45%) and Cyanobacteria (0.73% *vs* 0.51%) were decreased. The Firmicutes:Bacteroidetes ratio was 0.82 in the CTLs group, but 1.38 in IBD patients (Fig. 1a).

In CD patients only Bacteriodetes (32.83% *vs* 47.45%), Proteobacteria (13.67% vs 6.54%), Verrucomicrobia (3.76% *vs* 1.02%) and Cyanobacteria (0.38% vs. 0.73%) showed the same evidence of variation between the groups, and in UC patients only Proteobacteria did not attain the same significant difference. Additionally, in this group, Actinobacteria phylum (8.42% *vs* 2.26%) was significantly increased compared to CTLs (Fig. 1b,c).

**Figure 2:**
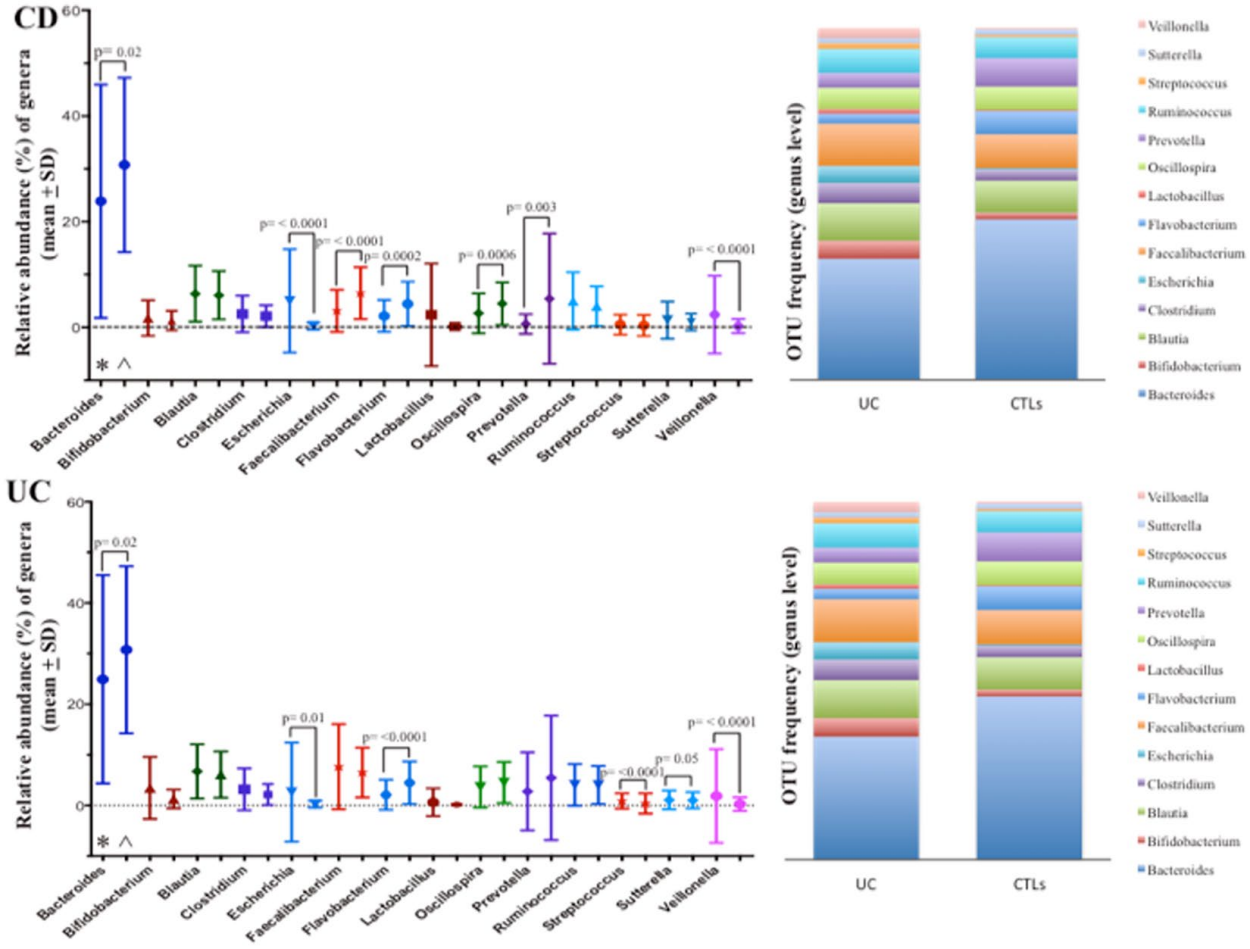
Microbiome taxonomic composition at genus level in CD, UC and controls subjects. Relative abundance of genera and OTU frequency are shown in CD (*) and UC (*) patients compared to controls (CTLs, ^). Significant differences with p < 0.05 are shown.

The relative abundances and frequency of the OTUs at order (Supplementary 1, Figs S1,2) and genus level (Supplementary 1, Fig. S3) are shown. At genus level, *Escherichia*, *Faecalibacterium*, *Streptococcus*, *Sutterella* and *Veillonella* all significantly increased in the IBD group, whereas *Bacteroides*, *Flavobacterium*, and *Oscillospira* geni all decreased. In CD patients, only *Escherichia* and *Veillonella* significantly increased, and, among the genera that decreased, also *Prevotella* decreased significantly. Interestingly, in this group the *Faecalibacterium* genus decreased compared to the control group, as opposed to what was observed in IBD as a whole and in the UC group. In UC patients, increase of the *Faecalibacterium* and decrease of the *Oscillospira* genera, respectively, were not statistically significant (Fig. 2).

**Figure 3:**
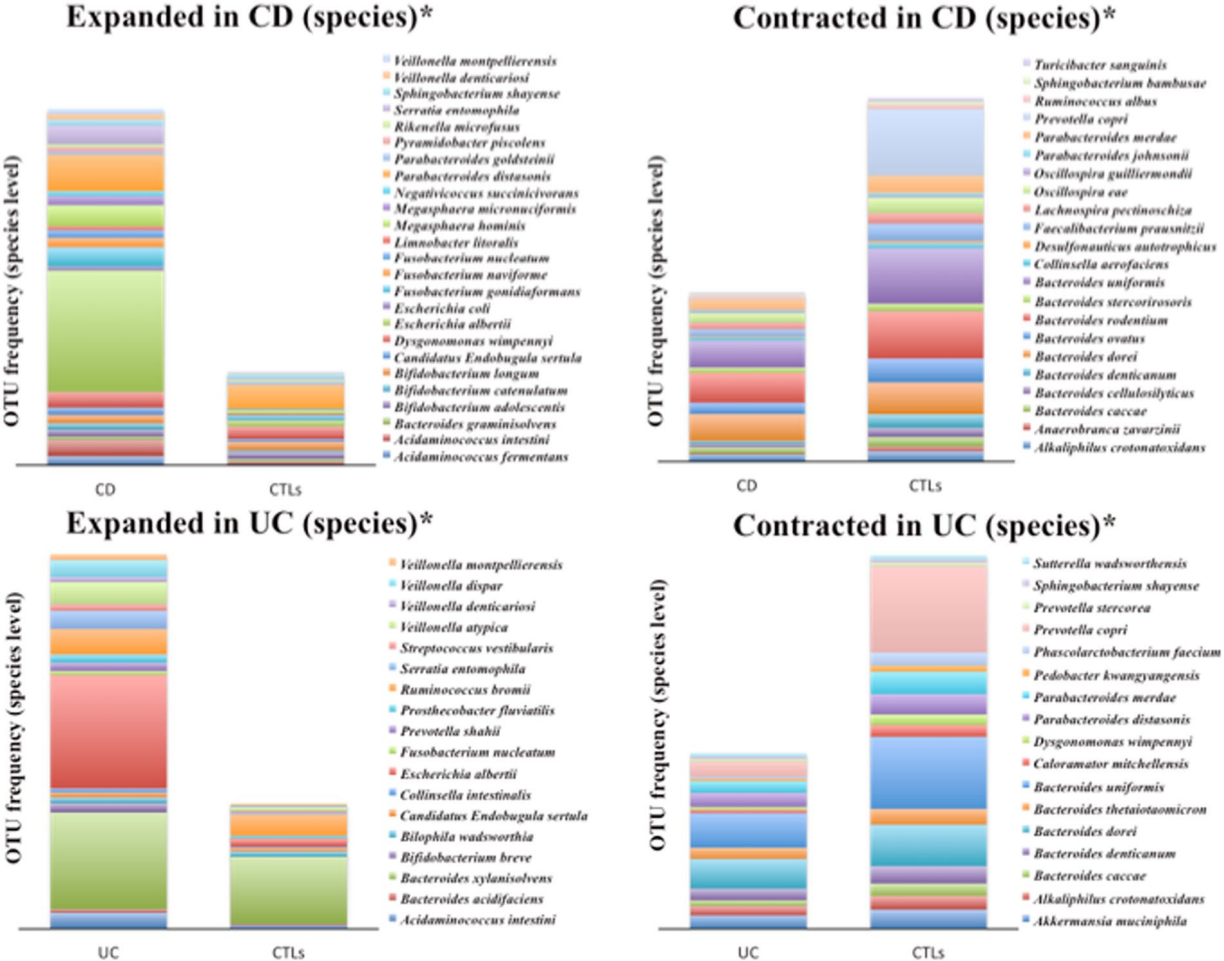
Relative abundance of species in CD e UC patients compared to controls subjects. OTU frequency of species higher than 0.01% are indicated as expanded or contracted in patients groups and controls subjects. *Indicates that only levels of significance with p < 0.05 for the species indicated are shown.

Finally, in Fig. 3 the distribution of the OTUs at the species level is shown for those that appeared increased and decreased in the CD and UC groups, respectively. In the CD group, 25 species increased and 22 species decreased, respectively, whereas in the UC group, 18 species increased and 17 species appeared reduced, respectively. Amongst Proteobacteria, *Escherichia alberti* resulted much more abundant than in controls (4.61% *vs* 0.24% in CD; 3.28% *vs* 0.24% in UC), and, among Bactroidetes, *Prevotella copri* was the main species reduced (0.02% *vs* 3.91% in CD; 0.68% vs 3.91% in UC) in both patient groups.

Neither the diet nor the activity of disease did affect the microbiome composition. Regarding the localization of the disease, only the relative abundance of Fusobacteria correlated with the colon *vs*. the ileum localization of the Crohn’s disease. Finally, a significant correlation between abundance of Firmicutes and Verrucomicrobia and medications was observed in CD group, and between Actinobacteria and therapy in the UC group (see Supplementary 1, Table S1a,b,c).

### Metabolomics analysis

#### GC-MS analysis

**Figure 4:**
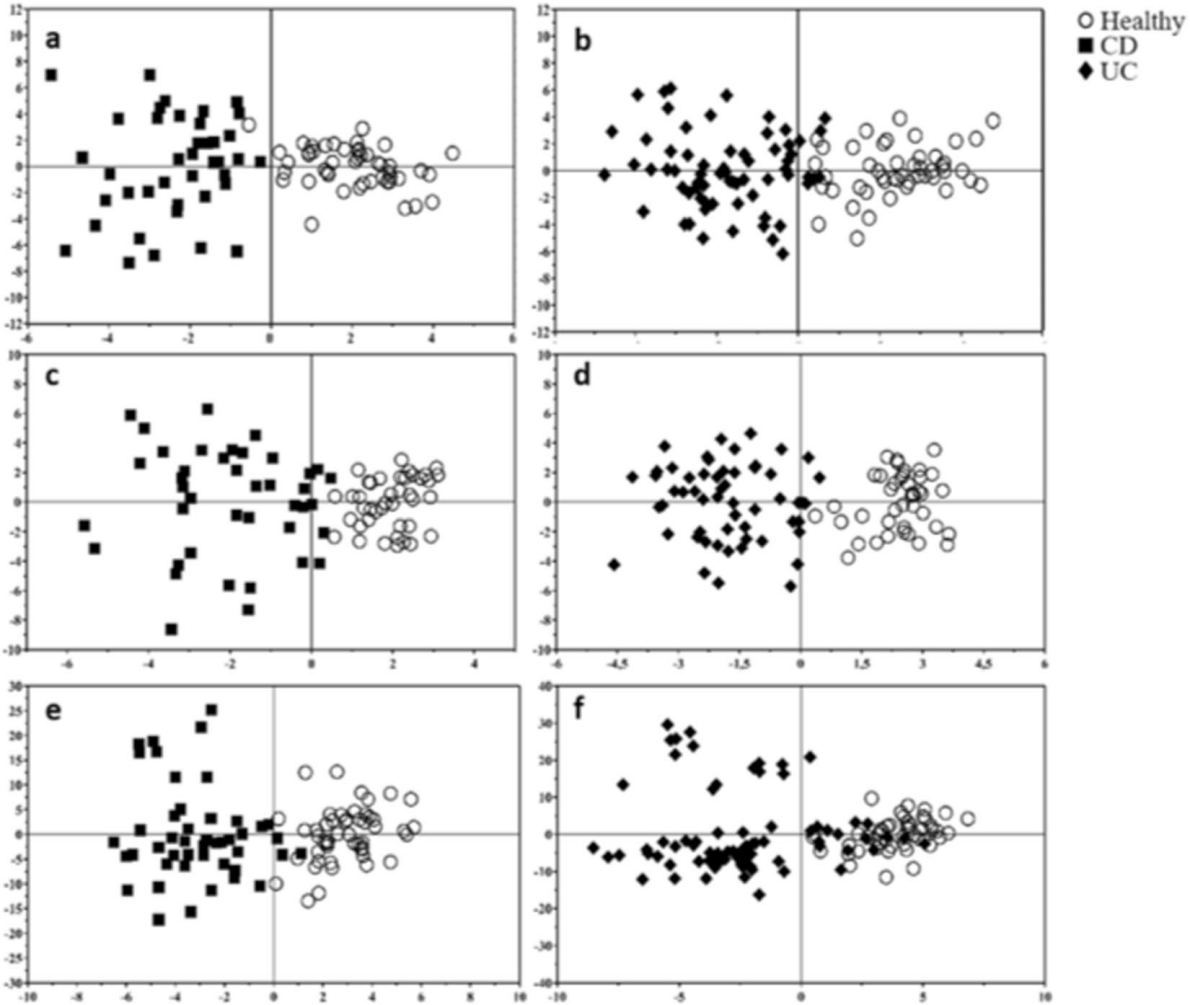
OPLS-DA score plots. In the first column CD vs healthy comparisons are shown while the second column of plots contains UC vs healthy comparisons. Plots were obtained with GC-MS (**a,b**), ^1^H-NMR (**c,d**) and LC-MS/MS QTOF analysis (**e,f**).

The OPLS-DA score plot demonstrates clear separation between both CD patients and healthy controls and UC patients and healthy controls (Fig. 4a,b). The quality of the models was evaluated using the corresponding Partial Least Square Discriminant Analysis (PLS-DA) models using a 7-fold cross-validation and permutation test over 400 times (Supplementary 1, Table S2). Moreover, to investigate if we could distinguish between the two pathological conditions, an OPLS-DA model was created. This comparison did not show good results, indicating an intrinsic similarity in metabolic profiles (Supplementary 1, Table S1). Discriminant metabolites were highlighted by the means of an S-plot and a Mann-Whitney test was carried out to find significant differences between the classes.

**Figure 5:**
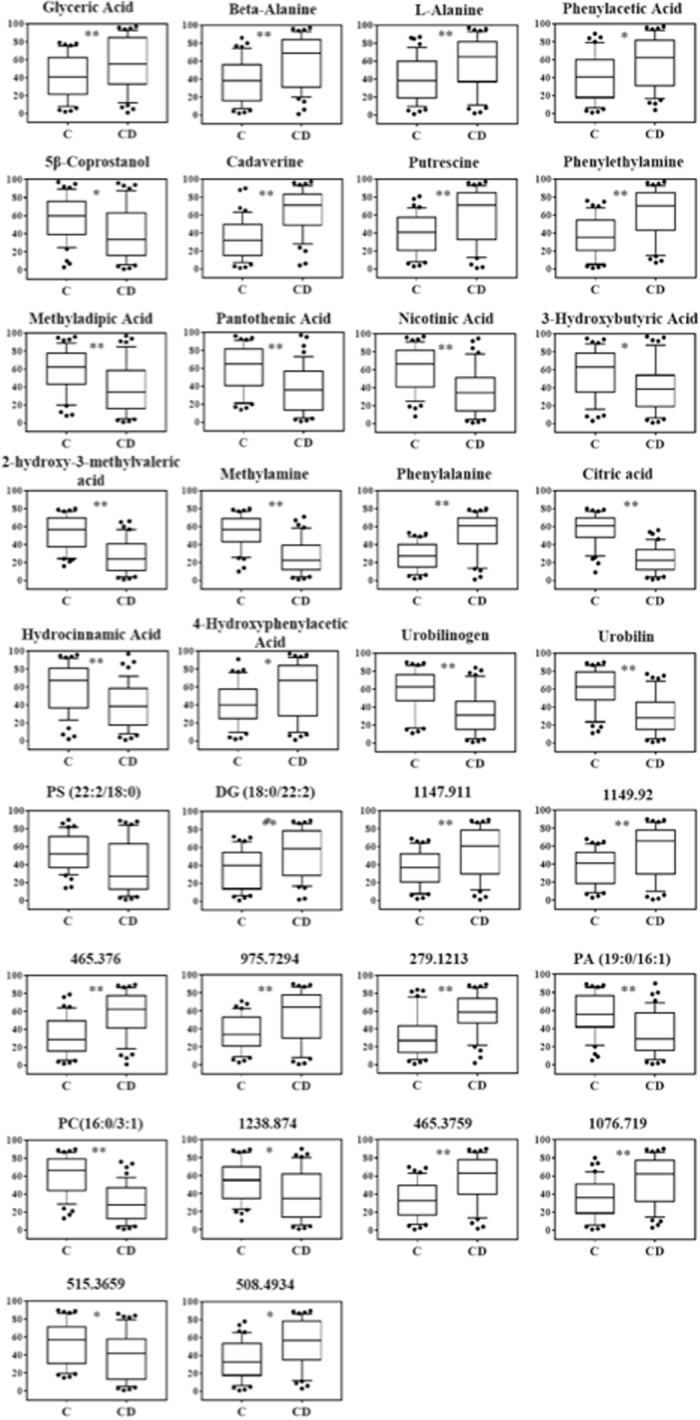
Statistically significant metabolites in CD vs healthy comparison. Discriminant metabolites obtained with the MVA, underwent to a Mann-Whitney test to determine which metabolites were statistically significantly variated. The resulted metabolites obtained are shown. Relative concentrations are represented in the y axis. * And **Indicates levels of significance with p < 0.05 and <0.01, respectively.

Fourteen metabolites were detected to be responsible for the separation of the CD patients from the healthy control group (Fig. 5). Of these, eight were significantly increased in CD patients (alanine, beta-alanine, phenylacetic acid, 4-hydroxyphenylacetic acid, glyceric acid, phenylethylamine, putrescine and cadaverine) and six metabolites significantly decreased in CD patients (nicotinic acid, pantothenic acid, 3-methyladipic acid, 5β-coprostanol, 3-hydroxybutyric acid and hydrocinnamic acid).

**Figure 6:**
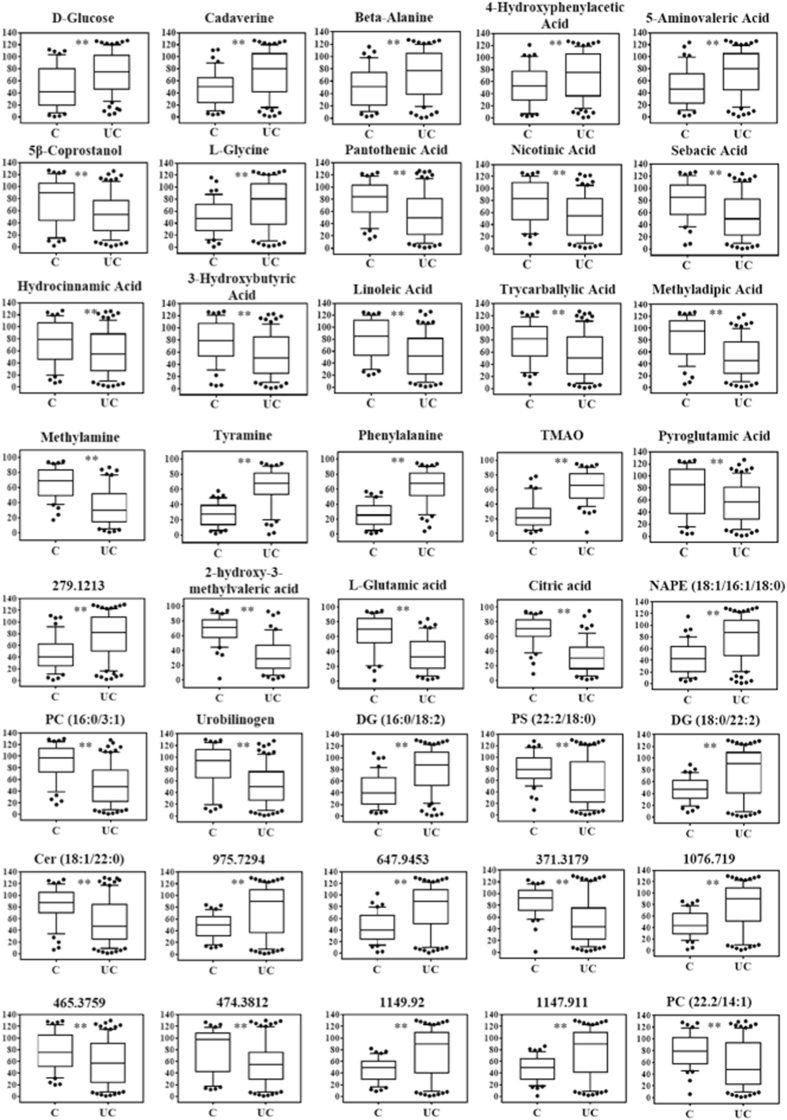
Statistically significant metabolites in UC vs healthy comparison. Discriminant metabolites obtained with the MVA, underwent to a Mann-Whitney test to determine which metabolites were statistically significantly variated. The resulted metabolites obtained are shown. Relative concentrations are represented in the y axis. * And **Indicates levels of significance with p < 0.05 and <0.01, respectively.

Regarding the comparison between UC patients and healthy controls, sixteen metabolites were able to discriminate between the two groups (Fig. 6), nine of which were in common with the CD patients-healthy controls comparison. Six metabolites significantly, 4-hydroxyphenylacetic acid, glucose, cadaverine and 5-aminovaleric acid), while 10 metabolites significantly decreased in UC patients (nicotinic acid, pantothenic acid, 3-methyladipic acid, pyroglutamic acid, 5β-coprostanol, 3-hydroxybutyric acid, hydrocinnamic acid, linoleic acid, sebacic acid and tricarballylic acid).

#### ^1^H-NMR analysis

Spectral resonances were assigned to individual metabolites on the basis of data published in the literature^12,13^ and using the 500 MHz library from Chenomx NMR suite 7.1 (Chenomx Inc., Edmonton, Alberta, Canada). OPLS-DA score plots demonstrated good separation between the healthy control group and CD and UC patients, respectively (Fig. 4c,d). However, just as with GC-MS data OPLS-DA was not able to distinguish between the two pathological conditions (UC and CD), indicating an intrinsic similarity in metabolic profiles (Supplementary 1, Table S2). In order to understand the actual trend of the metabolites, their relative concentrations were determined using the Chenomx NMR suite 7.1. The CD patients had a lower content of 2-hydroxy-3-methylvaleric acid, citric acid, and methylamine, but a higher content of cadaverine, putrescine and phenylalanine than the healthy controls (Fig. 5). The UC patients had significantly lower content of 2-hydroxy-3-methylvaleric acid, glutamic acid, citric acid, methylamine, but a higher content of cadaverine, trimethylamine-N-oxide (TMAO), tyramine and phenylalanine than the healthy controls (Fig. 6).

#### LC-QTOF-MS analysis

Peaks were identified and attributed to endogenous metabolites that included steroids, ceramides (Cer), phosphatidylserines (PS), phosphatidylcholine (PC), phosphatidylethanolamine (PE), diacylglycerols (DG), triacylglycerol (TG), n-acylphosphatidylethanolamines (NAPE) and other lipids.

OPLS-DA analysis displayed a clear separation between healthy subjects and both pathological classes (CD and UC) (Fig. 4e). As well as GC-MS and ^1^H-NMR analysis, the comparison between the two pathological classes did not show good separation (Supplementary 1, Table S2).

Seventeen metabolites were interpreted to be different between CD patients and healthy controls (Fig. 5). Using MS/MS fragmentation data, we were able to annotate six of them. DG (18:0/22:2) significantly increased in CD patients, while urobilin, PC (16:0/3:1), urobilinogen, PA (19:0/16:1) and PS (22:2/18:0) were found to be decreased.

Comparing UC patients with healthy controls, sixteen metabolites differentiated between the two classes. Nine of these were in common with the CD patients-healthy controls comparison. In particular, DG (16:0/18:2), DG (18:0/22:2) and NAPE (18:1/16:1/18:0) were identified to be significantly increased in UC patients feces, while PC (16:0/3:1), urobilinogen, PC (22.2/14:1) and Cer (18:1/22:0) were decreased in UC patients (Fig. 6).

Overall, neither the diet, nor the therapy, or the localization of the disease have had significant effect on the metabolome composition. In fact, the PLS-DA analysis for both UC and CD groups provided poor statistically significant results (see Supplementary 1, Table S3).

#### Pathways analysis

**Figure 7:**
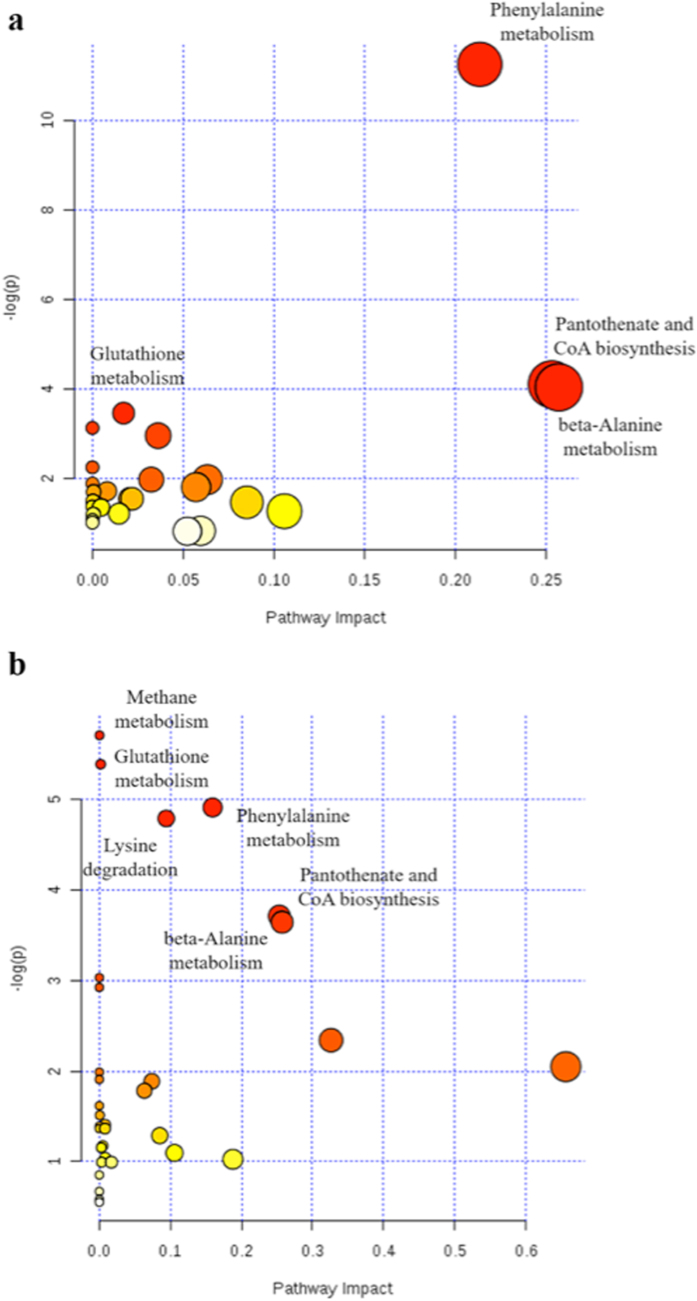
Relevant metabolic pathways involved in CD (**a**) and in UC (**b**).

Metabolic pathways were built using the MetaboAnalyst version 3.0^14^; only metabolites with significantly different (p < 0.05) concentrations between healthy patients and patients with CD and UC were used. The analysis showed how CD and UC significant metabolites were involved in different pathways as phenylalanine, glutathione and beta-alanine metabolism and pantothenate and CoA biosynthesis. The first three pathways are part of the amino acids metabolism while the last is involved in the metabolism of cofactor and vitamins. Moreover, the analysis demonstrated as UC metabolites were also implicated in methane metabolism and lysine degradation (Fig. 7a,b). The first is again comprised in the amino acids metabolism while the latter is classified as a part of the energy metabolism.

### Correlation between metabolome and microbiome

The observation was restricted to the microbial genera whose relative abundance resulted statistically different among the different groups of subjects and to metabolites that were characterized by the highest discriminative power among the patients and healthy controls (Supplementary 1, Fig. S4).

**Figure 8:**
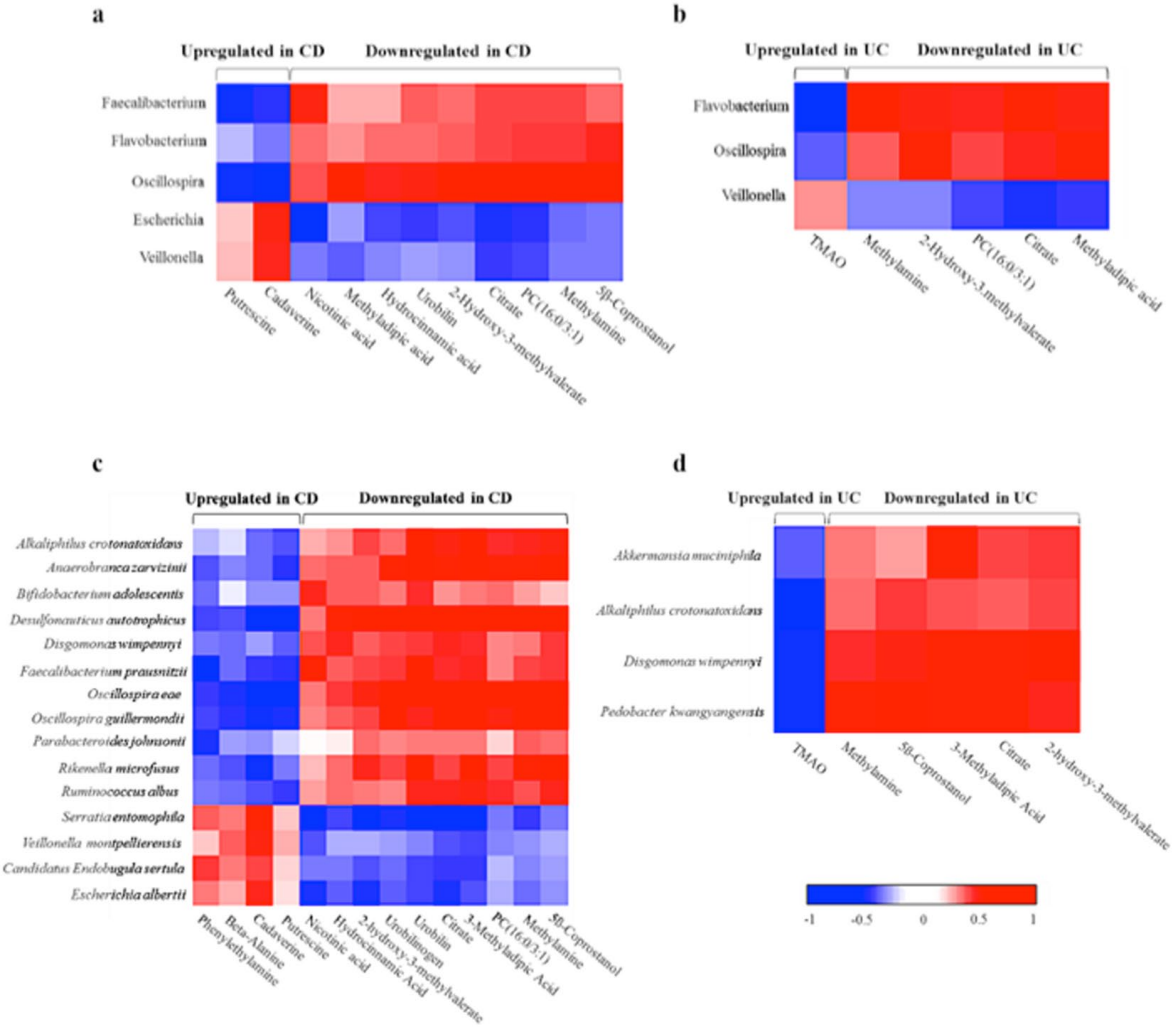
Inter-omic Spearman rank correlation between metabolites and bacterial genera and species. Spearman correlation between statistically different metabolites and bacterial genera was calculated both for CD (**a**) and UC (**b**). Spearman correlation between statistically different metabolites and bacterial species was calculated both for CD (**c**) and UC (**d**). Correlations with an r coefficient >0.5 are shown.

The Spearman correlation analysis revealed a strong association between five bacterial genera with 10 discriminant metabolites in CD patients (Fig. 8a). The most correlated genus was *Oscillospira*, particularly with hydrocinnamic acid, 3-methyladipic acid, 5β-coprostanol, citric acid, methylamine, 2-hydroxy-3-methylvaleric acid, PC (16:0/3:1) and urobilin (all positive correlations), while the genus negatively correlated with cadaverine and putrescine. In addition, the *Faecalibacterium* genus negatively correlated with the two biogenic amines, cadaverine and putrescine, while only nicotinic acid positively correlated. The *Escherichia* genus negatively correlated with nicotinic acid, citric acid and PC (16:0/3:1) and positively correlated with cadaverine. Lastly, a positive correlation was detected between the *Flavobacterium* genus and 5β-coprostanol and between the *Veillonella* genus and cadaverine.

The Spearman correlation coefficient was less strong for UC than for CD patients (Fig. 8b). Only three bacterial genera correlated with six metabolites. Particularly, a strong positive correlation was observed between *Flavobacterium* genus and 3-methyladipic acid, 2-hydroxy-3-methylvaleric acid, citric acid, methylamine and PC (16:0/3:1), while this genus negatively correlated with trimethylamine-N-oxide (TMAO). UC patients also presented correlations between *Oscillospira* genus and 3-methyladipic acid, 2-hydroxy-3-methylvaleric acid and citric acid, and between *Veillonella* genus and citric acid.

Fifteen bacterial species showed a good correlation with 14 discriminant metabolites in Crohn disease patients (Fig. 8c, and Supplementary 1, Fig. S5). Seven species, *Faecalibacterium prausnitzii, Oscillospira eae, Oscillospira guillermondii, Anaerobranca zavarzinii*, *Veillonella montpellierensis*, *Ruminococcus albus* and *Alkaliphilus crotonatoxidans* belonging to the Firmicutes phylum, four, *Desulphonauticus Autotrophicus, Serratia entomophila*, *Escherichia albertii and Candidatus Endobugula sertula* to the Proteobacteria phylum, three, *Dysgonomonas wimpennyi, Rikenella microfusu*s and *Parabacteroides johnsonii* to the Bacteroidetes phylum and finally only one, *Bifidobacterium adolescentis* to the Actinobacteria phylum. In particular, a strong positive correlation was seen between *Oscillospira eae* and 5β-coprostanol, 3-methyladipic acid, citric acid, methylamine, 2-hydroxy-3-methylvaleric acid, PC (16:0/3:1) and urobilin; between *Oscillospira guillermondii* and 5β-coprostanol, methylamine and PC (16:0/3:1); and finally between *Desulphonauticus autotrophicus* and 5β-coprostanol, 3-methyladipic acid, citric acid, methylamine and PC (16:0/3:1). On the other hand, a strong negative correlation between *Faecalibacterium prausnitzii* and phenylethylamine and between *Desulphonauticus autotrophicus* and putrescine and cadaverine was documented.

Evaluation of the metabolome-species relationship in UC patients showed good correlations between *Pedobacter kwangyangensisvs* and *Dysgonomonas wimpennyi* and 3-methyladipic acid, 5ß-Coprostanol, 2-hydroxy-3-methylvaleric acid, citric acid and methylamine, while only 3-methyladipic acid positively correlated with *Akkermansia muciniphila* species. A negative correlation was detected between TMAO and *Pedobacter kwangyangensisvs, Dysgonomonas wimpennyi* and *Alkaliphilus crotonatoxidans* species (Fig. 8d).

## Discussion

It has been established that the gut microbiota is dysregulated in IBD, which leads to the modification of the bacterial metabolic activity^15^. In the clinical management of IBD, there are significant, inherent challenges in elucidating whether dysbiosis contributes to disease pathogenesis or whether, by contrast, it could be a secondary change associated with the inflammatory process, which may be driven by host genetics and environmental factors like diet.

In this study, we have applied a dual-omics approach to improve our current understanding of IBD, and in particular the host-microbial interactions, and the functional aspects of the gut microbiota.

Whereas it has been reported previously in IBD that there is a decrease in Firmicutes and Actinobacteria abundance, along with high levels of Proteobacteria compared to healthy subjects^16^, we did not find this in our patient cohort apart from the increase in Proteobacteria. Our cohort demonstrated, instead, increased levels of Firmicutes in IBD (with statistical significance only in the UC group) and Actinobacteria (with statistical significance in UC patients). Since in our analysis the relative abundance of Firmicutes and Actinobacteria also correlated with the IBD therapy, this aspect should be further investigated.

The bacterial complexity was significantly lower in IBD, CD and UC patients compared to control subjects, as previously reported. Taken together, these data may reflect at least in part the results obtained from the metabolomics analysis, which revealed characteristic clustering profiles among healthy subjects and patient categories (IBD, CD, and UC). Two biogenic amines, cadaverine and putrescine were found significantly increased in CD patients compared to healthy subjects. Polyamines are formed mainly by the decarboxylation of certain amino acids or by transamination of aldehydes or ketones, and are produced both by the host and by intestinal flora bacteria. While their specific role is still largely unknown, polyamines are involved in numerous physiological processes such as the preservation of membrane integrity and nucleic acids metabolism. Furthermore, they have a crucial role in the regulation of gene expression and translation^17^. Several studies demonstrated how polyamines are necessary for the division of epithelial cells^18–21^. In fact, the normal growth of the intestinal mucosa depends on the polyamines availability within the cell division crypts, and such substrates could be synthesized endogenously or absorbed at the lumen level. Elevated levels of polyamines seem to have a toxic effect and are associated with several diseases. It is believed that at the basis of this toxicity there is the oxidative stress caused by polyamines catabolism^22^. In our study, the Spearman correlation analysis demonstrated that the increase of the two polyamines negatively correlated with the amount of two bacterial genera belonging to the Firmicutes phylum, *Faecalibacterium* and *Oscillospira*. Moreover, cadaverine levels were also directly associated with the abundance of another Firmicutes genus, *Veillonella*, and the Proteobacterium *Escherichia*. Interestingly, *Oscillospira* and *Faecalibacterium* genera seems to have a protective action against inflammation^23^.

The increase of glyceric acid in the stool of patients with CD could be due to a release of triacylglycerols associated with the colon mucosa^24^. The high levels of amino acids, such as alanine, beta-alanine and phenylalanine in the feces of CD patients might, in part, result from malabsorption due to inflammation in these patients, or reflect an increase of the producing bacteria.

Two group B vitamins, nicotinic acid and pantothenic acid, significantly decreased in feces of CD patients^25^. Some studies report that pantothenic acid may have a protective effect against oxidative stress in mammalian tissues^26^. It seems that nicotinic acid might also have a protective effect on the mucosa of the colon, reducing inflammation^27^. The decreased levels of nicotinic acid in patients with CD may be due to a smaller presence of nicotinic acid producing bacteria. Decreased levels of this vitamin directly correlated with the reduced amount of the *Faecalibacterium* bacteria, particularly, with the decreased abundance of *Faecalibacterium prausnitzii*. It has already been demonstrated that *Faecalibacterium* has a protective role against inflammation of the colon mucosa^28^. *Faecalibacterium prausnitzii* abundance also inversely correlated with phenylethylamine. This amino acid is biosynthesized from the amino acid L-phenylalanine by enzymatic decarboxylation via the enzyme aromatic L-amino acid decarboxylase^29^. In addition to its presence in mammals, phenethylamine is found in many other organisms and foods, such as chocolate, especially after microbial fermentation. The concentration of phenylethylamine increased in feces of CD patients, suggesting that the depletion of *Faecalibacterium prausnitzii* could lead to an increase of some amine, which in turn could play a role in the pathogenesis of an inflammatory process^30^.

The amount of methylamine decreased in the aqueous fecal water extracts of patients with CD. This compound is derived from intestinal degradation of food components such as choline and carnitine by microbiota. The depletion of this microbiota-related metabolite correlated with the decrease of *Oscillospira* amount in feces, confirming the perturbation of the microbial homeostasis in patients with CD^31^.

In our study, 5β-coprostanol decreased in feces of CD patients. 5β-coprostanol derives from the catabolism of cholesterol by gut microbiota^32^. Again, the reduced amount of this metabolite correlated with the *Oscillospira* and *Flavobacterium* decrease in feces of CD patients. The low abundance of *Oscillospira* genus bacteria correlated also to the reduced levels of hydrocinnamic acid, 3-methyladipic acid, citric acid and 2-hydroxy-3-methylvaleric acid, confirming again the importance of this bacterium in the gut metabolism and wellness.

As several studies demonstrated, diacylglycerols, such as DG (18:0/22:2), are involved in the activation of the protein kinase C (PKC) in many cell types^33,34^. The upregulation of DG (18:0/22:2) in CD could suggest a modification of the protein kinase C (PKC) pathway. PKC plays a crucial role in many aspects of the gastrointestinal tract homeostasis, taking part in many physiological and pathological processes such as development, inflammation and tumorigenesis^35^.

Several others lipids, such as urobilin, urobilinogen, PC (16:0/3:1), PA (19:0/16:1) and PS (22:2/18:0), were found to be decreased in the same patients. Urobilinogen and urobilin are open tetrapyrroles deriving from bilirubin catabolism by gut microorganisms and are excreted with urines and feces^36^. The urobilin level reduction might be due to a significantly lower conversion rate of urobilin in CD patients as compared to the controls and this could indicate an altered entero-metabolic function in these patients^37^. The downregulation of phosphocholines, such as PC (16:0/3:1), could reflect a defect of PC synthesis or secretion. It has been known that several pathologies, like IBD or cancer, are correlated to the phospholipids pathways^38^. Cytosolic phospholipase A2 is activated by low concentration of Ca^2+^, and during this activation the lipase is transferred from the cytosol to the cell membrane to initialise the arachidonic acid cascade producing many inflammatory mediators^39^.

In addition to the previously described metabolites, fecal extracts derived from UC patients showed an increase in tyramine, TMAO, glycine, glucose, 5-aminovaleric acid, DG (16:0/18:2) and NAPE (18:1/16:1/18:0), and a decrease in glutamic acid, pyroglutamic acid, linoleic acid, sebacic acid and trycarballylic acid, PC (22.2/14:1), and Cer (18:1/22:0). Furthermore, in UC patients there wa﻿s a﻿ decrease in 3-methyladipic acid, 2-hydroxy-3-methylvaleric acid, citric acid, methylamine and PC (16:0/3:1). The reduced levels of all these metabolites strongly correlated with the lower abundance of the *Flavobacterium* genus, belonging to the Bacteroidetes phylum. This genus also negatively correlated with TMAO levels. TMAO is generated by anaerobic bacteria through the digestion of dietary phosphatidylcholine and carnitine in a microbial-mammalian co-metabolic pathway. One previous study has found that plasma TMAO levels were significantly lower in UC population, suggesting that TMAO may be clinically useful in monitoring UC patients^40^.

3-Methyladipic acid, 2-hydroxy-3-methylvaleric acid and citric acid levels also positively correlated to *Oscillospira* abundance, suggesting a putative anti-inflammatory effect of *Oscillospira* in UC.

As previously described in the results, glucose increased in feces of UC patients. Glucose serves as an energy source for normal intestinal mucosa and its utilization is reduced during malnutrition and starvation, a common symptom observed in UC^41^. High levels of glucose thus indicate the inability of the colonic mucosal cells to utilise it for energy requirements.

The increased levels of NAPE (18:1/16:1/18:0) in UC patients suggest a modification of endocannabinoid system that has been demonstrated to be involved in the regulation of numerous gastrointestinal functions including gut homeostasis, modulating gastrointestinal motility, visceral sensation, and inflammation. Moreover, these metabolites have been recently implicated in IBD pathogenesis^42,43^. NAPEs are synthesized in the small intestine and after their synthesis are converted to the active NAEs. NAPEs are also converted into phosphatidic acid and anandamide by N-acylphosphatidylethanolamine-hydrolysing phospholipase D enzyme. Anandamide and 2-arachidonoylglycerol are endogenous bioactive lipids that bind and activate the cannabinoid receptors^44^. Decreased levels of anandamide and its synthesizing enzyme were found in the intestinal biopsies of UC patients^45^.

Our study combined the quantitative analysis of metabolites and microbiome in feces samples patients with CD and UC, and showed that this approach can be used to discriminate between healthy and diseased subjects. The results suggest that the detection of gut microbiota biomarkers in association with the comparative analysis of metabolites related to microbial metabolism or microbial-host co-metabolism could help to better understand the pathogenesis of IBD, and lead to the development of strategies for the early disease prediction and promote the development of novel targeted therapies.

## Methods

### Patients

The study was conducted in accordance with the principles of good clinical practice. The institutional ethics committee (Azienda Ospedaliero-Universitaria di Cagliari, Italy) approved the study, and written informed consent was obtained from each participant. All eligible UC (n = 82) and CD (n = 50) patients had their diagnosis confirmed by endoscopic, histological and radiographic data. Disease activity was verified by well-established criteria, including endoscopic grading according to the CDEIS and Rutgeerts scores for CD patients, and the Mayo score for UC patients^46^ (see Supplementary 1, Tables S5, S6). The healthy volunteers (n = 51) were recruited locally (Table 1). The exclusion criteria were as follows: (a) age >80 or <20 years; (b) use of antibiotics or probiotics in the last three months and (c) pregnancy. Each participant was given a sample collection kit with instructions. Hence, one fecal sample from each subject was collected and subsequently delivered to the laboratory within 3 hours. Samples were stored at −80 °C until use.

**Table 1:** Subjects used for analysis.

Groups		Healthy	Crohn Disease	Ulcerative Colitis
Age		40.7 ± 13	48.8 ± 13	47.3 ± 12
Sex	*Male*	31	24	44
	*Female*	20	26	38
Diet	*Mediterranean*	49	50	82
	*High-protein*	2	—	—
Smoke	*Yes*	18	12	8
	*Ex-smokers* (>*2 years)*	5	24	20
Alcohol	*Yes*	28	15	10
Coffee	*Yes*	44	26	51
Therapy	*Mesalazine/Salazopyrin/*	—	8	54
	*Steroids*			
	*Azatioprine*	—	9	15
	*Infliximab/Adalimumab*	—	27	9
	*No IBD therapy*	51	6	4
Lesion localization	*Ileum-colon*	—	19	—
	*Colon*	—	3	—
	*Ileum*	—	24	—
	*Ileum-caecum*	—	3	—
	*Rectum sigmoid*	—	1	21
	*Rectum*	—	—	16
	*Descending colon*	—	—	6
	*Rectum-sigmoid-descending colon*	—	—	14
	*Rectum-sigmoid-descending- transverse colon*	—	—	5
	*Pancolitis*	—	—	20
Total		51	50	82

### Gut microbiota analysis

#### DNA extraction and quantification

DNA extraction from thawed fecal samples was performed using the QIAamp DNA stool MiniKit according to the instructions of the manufacturer (Qiagen), with minor modifications (see Supplementary 1).

#### Real-time quantitative PCR

Quantitative PCR (qPCR) was performed using degenerate primers encompassing the V3 and V4 hypervariable regions of the bacterial 16 S rRNA gene (see Supplementary 1).

#### Library preparation and sequencing

Barcoded amplicon libraries for the analysis on the Illumina MiSeq platform were generated using degenerate primers targeting the V3 and V4 hypervariable region of the bacterial 16 S rRNA gene and Nextera XT index kit (Illumina) (see Supplementary 1).

#### Sequence processing and data analysis

Analysis of the data generated on the Miseq System was carried out using the BaseSpace 16 S Metagenomics App (Illumina), whereas operational taxonomic unit (OTU) mapping to the Greengenes database (V.13.8) were performed using the Quantitative Insights Into Microbial Ecology (QIIME) platform (V.1.8.0). Sequences containing ambiguous or low-quality bases were filtered out using QIIME filter53. Remaining sequences were assigned to each sample according to the unique barcodes. Alpha diversity was estimated using Shannon index metric.

### Metabolomics analysis

#### Stool samples extraction and preparation

Frozen feces (300 mg) were extracted with a solution of methanol\water (80:20) and the extract was divided in 3 aliquots for GC-MS^1^, H-NMR and LC-QTOF-MS analysis (see Supplementary 1).

#### Data processing

Chromatograms deriving from GC-MS and LC-QTOF-MS were processed to obtain a matrix of features present across all samples (see Supplementary 1).

#### Multivariate statistical analysis

Multivariate statistical data analysis was performed using SIMCA (version 14.0, Umetrics, Umea, Sweden). Principal components analysis (PCA) was used to identify the presence of outliers and to verify the influence of coffee, alcohol and smoke on metabolites composition of fecal samples. PCA is an unsupervised analysis that allows estimating and visualizing the distribution of the samples. Partial Least Square-Discriminant Analysis (PLS-DA) was performed to verify the influence of disease activity, disease localization and medications on metabolites composition. Orthogonal Partial Least Square-Discriminant Analysis (OPLS-DA) was performed in order to classify samples and elucidate metabolites able to differentiate the classes.

#### Univariate statistical analysis

GraphPad Prism software (version 7.01, GraphPad Software, Inc., CA, USA) was used to perform the univariate statistical analysis of the data and Spearman correlations between the microbiome and the metabolome. To verify the significance of metabolites obtained using multivariate statistical analysis and to find differences in the microbiome, the Kruskall-Wallis Mann and the Whitney test were performed.

### Availability of data and material

All Illumina sequence data and all microbiome-metabolome correlation data from this study are available from the corresponding author on reasonable request. The datasets (classification, number of reads, percentages, Spearman correlations) supporting the conclusions of this work are included within the article as additional files (Supplementary information 2 and 3).

### Ethical approval and consent to participate

The subjects gave informed consent, and the study protocol was approved by the Institution Ethics Committee (AOU Cagliari, Italy. Prot. NP/2014/3504; Prot. PG/2014/11480).

## Electronic supplementary material

Supplementary Information 1

Dataset 1

Dataset 2
