# Author Correction: Cross sectional evaluation of the gut-microbiome metabolome axis in an Italian cohort of IBD patients

**DOI:** 10.1038/s41598-018-23330-5

**Published:** 2018-03-19

**Authors:** Maria Laura Santoru, Cristina Piras, Antonio Murgia, Vanessa Palmas, Tania Camboni, Sonia Liggi, Ivan Ibba, Maria Antonia Lai, Sandro Orrù, Sylvain Blois, Anna Lisa Loizedda, Julian Leether Griffin, Paolo Usai, Pierluigi Caboni, Luigi Atzori, Aldo Manzin

**Affiliations:** 1 Department of Biomedical Sciences, University of Cagliari, Cagliari, Italy; 2 Department of Life and Environmental Sciences, University of Cagliari, Cagliari, Italy; 3 Department of Medical Sciences and Public Health, University of Cagliari and Gastroenterology Unit, University Hospital of Cagliari, Cagliari, Italy; 4 Gastroenterology Unit, University Hospital of Cagliari, Cagliari, Italy; 5 Consiglio Nazionale delle Ricerche (C.N.R.), Istituto di Ricerca Genetica e Biomedica (I.R.G.B.), Monserrato, Cagliari, Italy; 6 Department of Biochemistry, University of Cambridge, Cambridge, UK

Correction to: *Scientific Reports* 10.1038/s41598-017-10034-5, published online 25 August 2017

Sylvain Blois was omitted from the author list in the original version of this Article. This has been corrected in the PDF and HTML versions of the Article, and in the accompanying Supplementary Information file.

The Author Contributions section now reads:

A.M., L.A., P.U., and P.C. conceived the study, directed the project and designed the experiments. M.A.L. and I.I. obtained the samples and clinical details. T.C., V.P., S.O., and A.L.Z. performed microbiome experiments and data analysis. S.B. performed preliminary experiments for bacterial DNA quantitation. M.L.S., C.P., A.Mu., and S.L. performed metabolomics experiments and data analysis. M.L.S. and A.M. wrote the first draft of the manuscript, and L.A. and J.L.G. contributed to the final version. M.L.S., C.P., A.Mu., V.P., T.C., S.L., I.I., M.A.L., S.O., A.L.Z., J.L.G., P.U., P.C., L.A., and A.M. critically reviewed the data and the manuscript. All authors read and approved the final version of the manuscript.

