# Supplementary material for: Cross sectional evaluation of the gut-microbiome metabolome axis in an Italian cohort of IBD patients
Source: Sci Rep. 2017 Aug 25;7:9523. doi: 10.1038/s41598-017-10034-5 (PMC5573342; doi:10.1038/s41598-017-10034-5)
Supplement: Supplementary file 1 — Supplementary Information 1 [file 41598_2017_10034_MOESM1_ESM.pdf]

Cross sectional evaluation of the gut-microbiome metabolome axis in an Italian cohort of IBD patients

Maria Laura Santoru[1], Cristina Piras[1], Antonio Murgia[2], Vanessa Palmas[1], Tania Camboni[1], Sonia Liggi[1], Ivan Ibba[3], Maria Antonia Lai[4], Sandro Orrù[3], Sylvain Blois[1], Anna Lisa Loizedda[5], Julian Leether Griffin[6], Paolo Usai[3], Pierluigi Caboni[2], ^Luigi Atzori[1], *Aldo Manzin[1]

[1]Department of Biomedical Sciences, University of Cagliari

[2]Department of Life and Environmental Sciences, University of Cagliari

[3]Department of Medical Sciences and Public Health, University of Cagliari and [4]Gastroenterology Unit, University Hospital of Cagliari

[5]Consiglio Nazionale delle Ricerche (C.N.R.), Istituto di Ricerca Genetica e Biomedica (I.R.G.B.), Monserrato, Cagliari, Italy

[6]Department of Biochemistry, University of Cambridge, Cambridge, UK

*Corresponding Author: Prof. Aldo Manzin: aldomanzin@medicina.unica.it +39-70-675 4067

AL^ and MA* contributed equally to this work.

METHODS

Gut microbiota

DNA extraction and quantification: 100-200 mg of fecal sample was mixed with 1.4-2.8 mL ASL buffer in a 2 mL tube and vortexed until the sample was thoroughly homogenized. Lysis was carried out at a temperature of 95°C for 5-10 minutes. Finally, DNA was extracted according to the instruction of the QIAamp DNA stool MiniKit and eluted in 200 µL elution buffer provided in the kit. DNA concentration and purity were measured using a NanoDrop ND-1000 spectrophotometer (Thermo Fisher Scientific), and samples were stored at −80°C until further processing.

Real-time quantitative PCR. Primers: forward 5'-CCTACGGGNGGCWGCAG-3'; reverse 5'-GACTACHVGGGTATCTAATCC-3'). Genomic DNA from the *E. coli* ATCC25922 reference isolate was used to prepare the standard curve (1:10 dilutions from 70.5 to 0.0075 ng). The qPCR was performed using a CFX96 Touch Real-Time System instrument (Bio-Rad) using the Bio-Rad CFX manager software, version 3.1 (Bio-Rad). Briefly, 20 µl PCR mixture contained 10 µl of SsoAdvanced Universal SYBR Green Supermix (Bio-Rad), 8 pmol of each primer, and 5 µl of DNA elution were used for each reaction. qPCR reactions consisted of an initial denaturation step of 5 min at 98°C, followed by 40 cycles of 15 s at 95°C, and 20 s at 59°C. The specificity of each PCR was determined by the melting curve analysis where by the temperature from 65°C to 95°C was increased with 0.5°C increments.

Library preparation and sequencing. For amplification reactions, fusion degenerate primer 16SF (5'-<u>TCGTCGGCAGCGTCAGATGTGTATAAGAGACAG</u>CCTACGGGNGGCWGCAG-3') and 16SR (5'-<u>GTCTCGTGGGCTCGGAGATGTGTATAAGAGACAG</u>GACTACHVGGGTATCTAATCC-3') were used, with ligated overhang Illumina adapter consensus sequences as indicated above in the underlinedregion. Each PCR reaction was carried out on an Applied Biosystem 9700 thermal cycler (Life Technologies) with 5 µL PCR buffer 10X, 0.5 µM each primer, 1.5 µL $MgSO_4$ 50 mM, 1 µL dNTP 10 mM each, 0.2 µL Platinum *Pfx* DNA polymerase 5U/µL (Life Technologies), 12.5 ng total DNA template, and nuclease-free, DEPC-treated water to a total volume of 50 µL. PCR reactions were performed as follows: after an initial denaturing step at 95°C for 3 min, 35 cycles were carried out consisting of denaturation at 95°C for 30 sec, annealing at 52°C per 30 sec, and extension at 72°C for 30 sec. After 35 cycles, the reaction was completed with a final extension of 7 min at 72°C.

The 550 bp 16S amplicons were purified using 20 µL Agencourt AMPure XP magnetic beads (Beckman Coulter) according to the manufacturer's instructions. For the multiplexing barcode procedure, the Illumina Nextera XT Index kit with dual 8 bases indices were used. PCR reactions containing 25 µL of KAPA HiFi HotStart Ready Mix 2x, 5 µL of i5 and i7 index (Illumina) each, 5 µL of purified amplicons, 5 µL Nextera XT Index primer 2, and nuclease-free, DEPC-treated water to a final volume of 50 µL, were carried out on an Applied Biosystem 9700 thermal cycler. PCR reactions consisted of one cycle of 95°C for 3 min, followed by eight cycles of 95°C for 30 sec, 55°C for 30 sec, and 72°C for 30 sec, followed by a final extension cycle of 72°C for 5 min. The barcoded amplicons were then purified using Agencourt AMPure XT

magnetic beads (Beckman Coulter, Inc.) following the instructions of the manufacturer. Afterwards, the barcoded libraries were quantified using the Agilent High Sensitive DNA Kit (Agilent Technologies), and normalized to ensure an equal representation of the samples. The quality and the size of the pooled libraries were verified using Agilent DNA 1000 Analysis kit (Agilent Technologies) on the Agilent 2100 Bioanalyzer system (Agilent Technologies), and finally sequenced on the MiSeq platform using MiSeq v3 Reagent Kit (Illumina), with the adapter-ligated library PhiX v3 used as a control.

Metabolomics

Frozen feces (300 mg) were mixed with 800 µL of methanol containing succinic acid-2,2,3,3-d4 as an internal standard (Sigma-Aldrich, St. Louis, MO, USA) and 200 µL of Milli-Q water and then vortexed. After 30 min, samples were centrifuged at 14000 rpm for 10 min at 4°C. The supernatant was aliquoted as following: GC-MS analysis (300 µL), LC-QTOF-MS analysis (50 µL) and NMR analysis (650 µL). For GC-MS analysis 300 µL of each fecal extract were dried under vacuum with vacuum concentrator overnight and were derivatised with 50 µL of methoxyamine dissolved in pyridine (10 mg/mL) (Sigma-Aldrich, St. Louis, MO, USA). After 17 h 100 µL of N-Methyl-N-(trimethylsilyl)-trifluoroacetamide, (MSTFA, Sigma-Aldrich, St. Louis, MO, USA) were added and left at room temperature for one hour. Successively, samples were re-suspended in 400 µL of hexane (Sigma-Aldrich, St. Louis, MO, USA) and filtered with Acrodisc Syringe Filters with 0.45 mm PTFE Membrane (SIGMA, St. Louis, MO, USA). For LC-MS QTOF analysis 50 µL of fecal extract were transferred into an eppendorf tube and added to 316 µL methanol and 633 µL dichloromethane. Samples were then centrifuged for 10 min at 12000 rpm. After centrifugation, the supernatant was transferred to another eppendorf tube where 200 µL of water were added to induce phase separation. All samples were then centrifuged at 8000 rpm for 10 min. The resulting organic phase was dried under nitrogen, re-suspended in 300 µL methanol and then filtered with Acrodisc Syringe Filters with 0.45 mm PTFE Membrane (SIGMA, St. Louis, MO, USA) [**1**]. For NMR analysis, dried hydrophilic fecal extracts were re-dissolved with 650 µL 100 mM KH_2PO_4/D_2O buffer pH 7.2 (99,8%, Cambridge Isotope Laboratories Inc, Andover, USA) and added with 50 µL of internal standard solution 5 mM (sodium 3-trimethylsilyl-propionate-2,2,3,3,-d_4, TSP, 98 atom % D, Sigma-Aldrich, Milan, Italy). An aliquot of 650 µL was transferred to 5-mm NMR tubes.

GC-MS analysis. One microliter of derivatised sample was injected splitless into a 7890A gas chromatograph coupled with a 5975C Network mass spectrometer (Agilent Technologies, Santa Clara, CA, USA) equipped with a 30 m ×0.25 mm ID, fused silica capillary column, with a 0.25 µM TG-5MS stationary phase (Thermo Fisher Scientific, Waltham, MA, USA). The injector and transfer line temperatures were at 250°C and 280°C, respectively. The gas flow rate through the column was 1 ml/min. For fecal samples analysis the column initial temperature was kept at 60 °C for 3 min, then increased to 140°C at 7°C/min, held at 140°C for 4 min, increased to 300°C at 5°C/min and kept for 1 min. Identification of metabolites was

performed using the standard NIST 08, and GMD mass spectra libraries and, when available, by comparison with authentic standards.

^{1}H-NMR spectroscopy analysis. ^{1}H-NMR experiments were carried out using a Varian UNITY INOVA 500 spectrometer operating at 499.839 MHz for proton and equipped with a 5 mm double resonance probe (Agilent Technologies, CA, USA). ^{1}H-NMR spectra were acquired at 300K with a spectral width of 6000 Hz, a 90° pulse, an acquisition time of 2 s, a relaxation delay of 2 s, and 256 scans. The residual water signal was suppressed by applying a presaturation technique with low power radiofrequency irradiation for 2 s. ^{1}H NMR spectra were imported in ACDlab Processor Academic Edition (Advanced Chemistry Development, 12.01, 2010) and pre-processed with line broadening of 0.1 Hz, zero-filled to 64K, and Fourier transformed. Each spectrum was manually phased and baseline corrected. Chemical shifts were referred to the TSP single resonance at 0.00 ppm. The ^{1}H-NMR spectra were reduced into consecutive integrated spectral regions (bins) of equal width (0.01 ppm) corresponding to the region 0.50–8.66 ppm. The spectral region between 4.74 and 4.94 ppm was excluded from the analysis to remove the effect of variations in the presaturation of the residual water resonance. The integrated area within each bin was normalized to a constant sum of 100 for each spectrum in order to minimize the effects of variable concentration among different samples. The final data set was imported into the SIMCA-P+ program (Version 14.0, Umetrics, Sweden), mean-centered and Pareto scaled column wise.

LC-QTOF-MS analysis. An Agilent 1200 series LC-QTOF-MS was used with an ESI source, operating in the positive ion mode. The electrospray capillary, needle and shield potentials were set to 60 V, 5850 V, and 450 V respectively. Nitrogen at 48 mTorr and 375 °C was used as a drying gas. For the fecal extracts full-scan spectra were obtained in the ranges of 100-1500 amu, scan time of 0.20 amu, scan width of 0.70 amu, and detector at 1500 V. The organic layers were analysed by a Pheomenex Kinetek C18 column EVO (100A. 150x2.1 mm 5μ) (California, USA). The mobile phase consisted of: (A) 60% of 10 mM ammonium formate and 40% of acetonitrile with 0.1% formic acid (*v/v*) and (B) a 10 mM ammonium formate solution containing 90% of isopropanol, 10% of acetonitrile with 0.1% formic acid (*v/v*). The mobile phase was pumped at a flow rate of 250 μL/min programmed as follows: initially at 68% of A for 1.30 min, then subjected to a linear decrease from 68% to 3% of A in 30 min and was then brought back to the initial conditions in 10 min. Putative recognition of all detected metabolites was performed using the Metlin and the Lipid Maps databases, whereas the most statistically significant metabolites were subjected to further identification with the means of targeted MS/MS analysis. Data were collected in the same *m/z* range of the MS scan mode and collision energy was set at 30V. All the discriminant metabolites MS and MS/MS data are reported (Supplementary table S4).

Data processing.

The R library XCMS [**2**, **3**] was utilized for peak detection and retention time correction. Parameters utilized for peak deconvolution for GC-MS matrices were manually optimized, whereas those used for LC-MS matrices were optimised using the R library IPO [**4**]. Grouping of features into pseudospectra and annotation

of isotopes and adducts was performed using the standard parameters of the R library CAMERA [**5**]. The resulting matrices were processed using an in-house python script to eliminate signals present in the blanks, keep only the most abundant feature per molecule and modify all zeroes present in the matrix by inserting half of the minimum value found for a feature. After manual correction of the filtered matrix to eliminate internal standard and any possible remaining noise signal, median fold change normalization was performed using an in-house python script in order to compensate for sample dilution biases [**6**].

**Phyla**	**Active *vs.* inactive CD** (*p* value)	**Active *vs.* inactive UC** (*p* value)
Firmicutes	0,3099	0,8674
Bacteroidetes	0,9434	0,3410
Actinobacteria	0,1086	0,4597
Proteobacteria	0,6639	0,2723
Verrucomicrobia	0,2707	0,2584
Cyanobacteria	0,8820	0,6802
Fusobacteria	0,7204	0,3842

Table S1a Mann-Whitney test for Phyla abundance in active v*s.* inactive disease.

**Phyla**	**Colon vs Ileum CD** (*p* value)
Firmicutes	0,2912
Bacteroidetes	0,9747
Actinobacteria	0,3510
Proteobacteria	0,3830
Verrucomicrobia	0,6256
Cyanobacteria	0,3816
Fusobacteria	**0,0305**

Table S1b Mann-Whitney test for Phyla abundance in colon *vs.* ileum localization of the disease.

Phyla	CD therapy (*p* value)	UC therapy (*p* value)
Firmicutes	**0,0238**	0,3431
Bacteroidetes	0,6208	0,2377
Actinobacteria	0,8172	**0,0311**
Proteobacteria	0,0950	0,0916
Verrucomicrobia	**0,0217**	0,1381
Cyanobacteria	0,8980	0,3429
Fusobacteria	0,3275	0,2222

Table S1c Kruskall-Wallis test for Phyla abundance and medications.

	**OPLS-DA models**				**Permutation***	
	**GC-MS**					
**Groups**	**Components**[a]	**R_2Xcum**[b]	**R_2Ycum**[c]	**Q_2cum**[d]	**R_2 intercept**	**Q_2 intercept**
Healthy *vs* IBD	1P+1O	0.112	0.663	0.439	0.333	-0.228
Healthy *vs* CD	1P+1O	0.144	0.778	0.519	0.478	-0.215
Healthy *vs* UC	1P+1O	0.119	0.734	0.504	0.408	-0.239
CD *vs* UC	1P+1O	0.105	0.494	0.036	0.419	-0.201
	**^{1}H-NMR**					
Healthy *vs* IBD	1P+3O	0.495	0.754	0.626	0.188	-0.430
Healthy *vs* CD	1P+1O	0.314	0.730	0.645	0.231	-0.241
Healthy *vs* UC	1P+2O	0.458	0.805	0.688	0.181	-0.327
CD *vs* UC	1P+1O	0.202	0.440	0.140	0.257	-0.235
	**LC-MS/MS QTOF**					
Healthy *vs* IBD	1P+2O	0.429	0.504	0.393	0.270	-0.136
Healthy *vs* CD	1P+3O	0.429	0.786	0.433	0.587	-0.240
Healthy *vs* UC	1P+2O	0.451	0.620	0.494	0.345	-0.146
CD *vs* UC	1P+1O	0.369	0.311	0.0628	0.229	-0.056

Table S2 MVA parameters. [a] The number of Predictive and Orthogonal components used to create the statistical models.
[b,c] R_2X and R_2Y indicated the cumulative explained fraction of the variation of the X block and Y block for the extracted components.

[d] Q_2cum values indicated cumulative predicted fraction of the variation of the Y block for the extracted components.

[*] R_2 and Q_2 intercept values are indicative of a valid model. The Permutation test was evaluated on the corresponding partial least square discriminant analysis (PLS-DA) model.

	PLS-DA models			
**Groups**	**Components[a]**	**R_2Xcum[b]**	**R_2Ycum[c]**	**Q_2cum[d]**
Active vs inactive CD	2	0.1	0.308	-0.0931
Active vs inactive UC	2	0.147	0.369	-0.21
Medications influence CD	3	0.126	0.406	-0.21
Medications influence UC	3	0.107	0.29	-0.169
Ileal vs colon CD	2	0.115	0.58	-0,19

Table S3 MVA parameters. [a] The number of components used to create the statistical models.

[b,c] R_2X and R_2Y indicated the cumulative explained fraction of the variation of the X block and Y block for the extracted components.

[d] Q_2cum values indicated cumulative predicted fraction of the variation of the Y block for the extracted components.

Compound	Parent ion m/z	Rt (min)	Adduct	Product ion (m/z)	Δppm
**urobilin**	617.3350	1.3	$[M+Na]^{+}$	470<br>345	3
**PC(16:0/3:1)**	549.3461	1.4	neutral	184.0709 $[\text{Head group}]^{+}$	4
	279.7294	1.7			
**urobilinogen**	597.3670	2.2	$[M+H]^{+}$	472<br>347	3
	508.4934	6.3			
	647.9453	11.0			
	515.3659	11.6			
**DG(16:0/18:2)**	634.5398	12.3	$[M+NH4]^{+}$	313.2$[RCOO+58]^{+}$ (FA16:0)<br>337.5$[RCOO+58]^{+}$ (FA18:2)	1
**PA (19:0/16:1)**	688.5040	14.8	neutral	355 [RCOO+58] (FA19:0)<br>281 $[RCO]^{+}$ (FA19:0)	0
**PC (22.2/14:1)**	783.5768	14.9	neutral	393 $[RCOO+58]^{+}$ (FA22:2)<br>184.07 $[\text{Head group}]^{+}$	0
	371.3179	15.0			
	474.3812	15.5			
	465.3760	15.8			
**DG (18:0/22:2)**	641.5974	16.4	$[M+H2H_2O]^{+}$	267.0 $[RCO]^{+}$<br>341.0 $[RCOO+58]^{+}$	14
**PS (22:2/18:0)**	844.6053	16.5	$+ H^{+}$	341 [RCOO+58] (FA18:0)<br>319 $[RCO]^{+}$ (FA22:2)	1
	1238.874	20.8			
**Cer (18:1/22:0)**	622.6167	22.7	$+H^{+}$	282 (FA18:1)<br>339 [RCOO+58] (FA18:1)<br>265 $[RCO]^{+}$ (FA18:1)	0
	1147.911	23.2			
	975.7294	23.2			
	1149.920	23.2			
	1076.719	23.5			
	465.3796	24.0			
**NAPE (18:1/16:1/18:0)**	981.7768	25.9	neutral	265 $[RCO]^{+}$ (FA18:1)<br>341 $[RCOO+58]^{+}$ (FA18:0)	0
	465.3759	29.0			

Table S4 Summary of discriminant compound identified by METLIN and Lipid maps databases and confirmed with MS/MS analysis.

Crohn’s Disease		
Surgery not needed		39
	Quiescent/remission	12
	Mild	5
	Moderate	6
	Severe	16
Surgery needed		11
	Quiescent/remission	4
	Mild	0
	Moderate	1
	Severe	2
Phenotype	Fistulising	9
	Inflammatory	28
	Stenosis	13
Total		50

Table S5 Classification of disease activity. Endoscopic grading of patients that did not need surgery was made according to the CDEIS score, while for patients that underwent surgery the Rutgeerts score was used.

Ulcerative Colitis		
Surgery not needed	Quiescent/remission	43
	Mild	15
	Moderate	17
	Severe	7
Total		82

Table S6 Classification of disease activity. Endoscopic grading of patients was made according to the Mayo score.

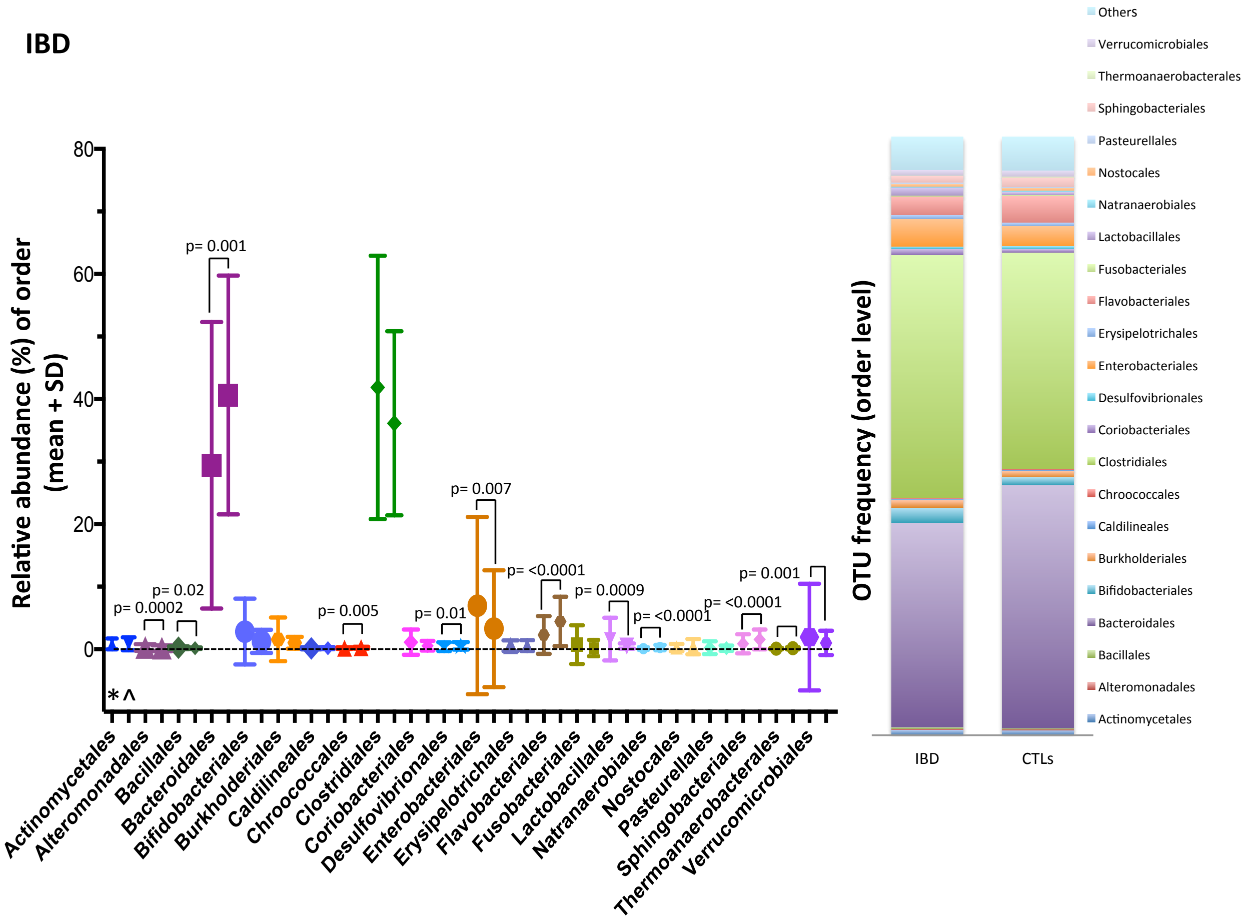

Figure S1 Microbiome taxonomic composition at order level in IBD patients and control subjects (CTLs). Relative abundance of orders and OTU frequency are shown. Significant differences with $p < 0.05$ are shown. *= patients; ^= controls.

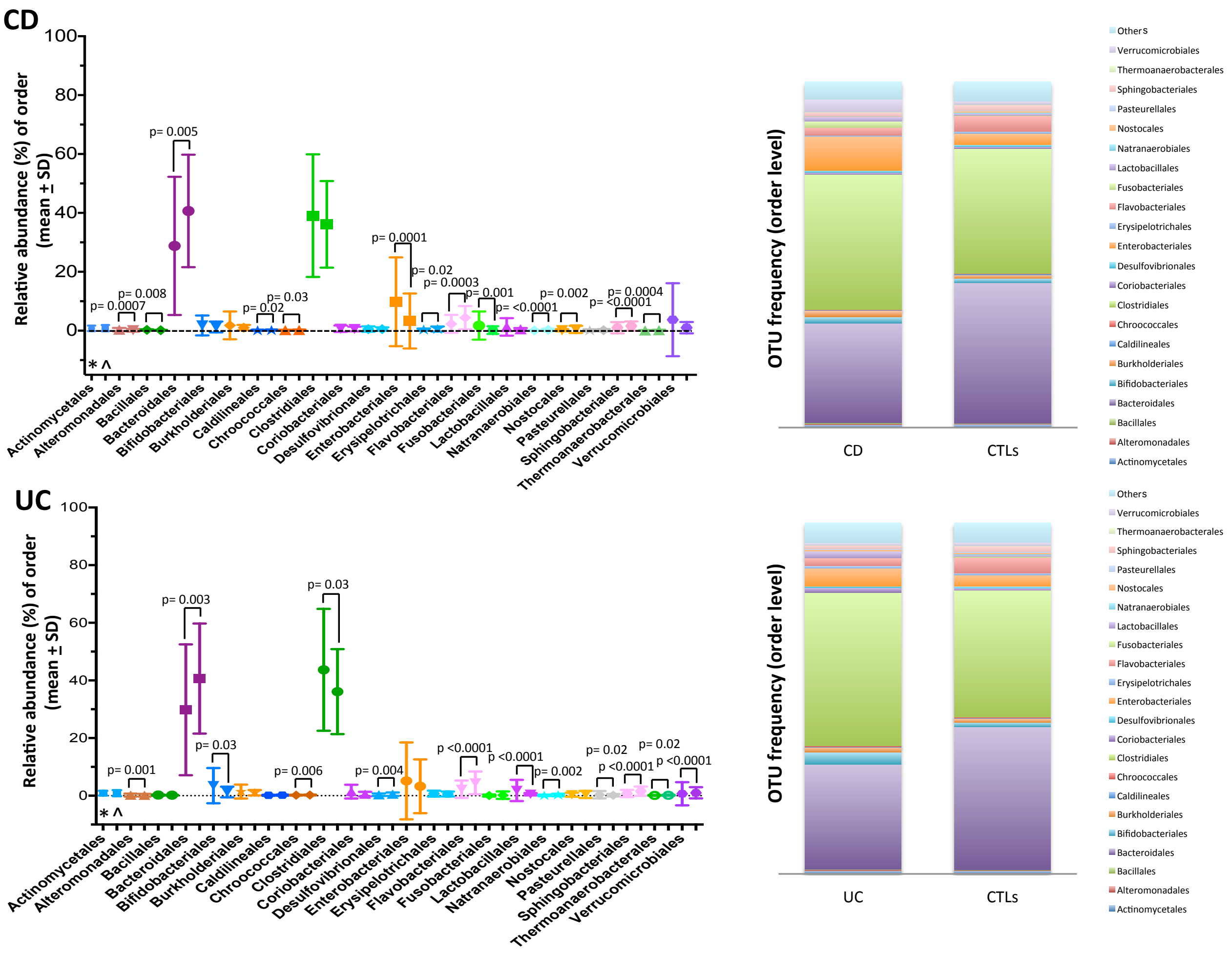

Figure S2 Microbiome taxonomic composition at order level in CD, UC and controls subjects. Relative abundance of orders and OTU frequency are shown in CD (*) and UC (*) patients compared to controls (CTLs, ^). Significant differences with p <0.05 are shown.

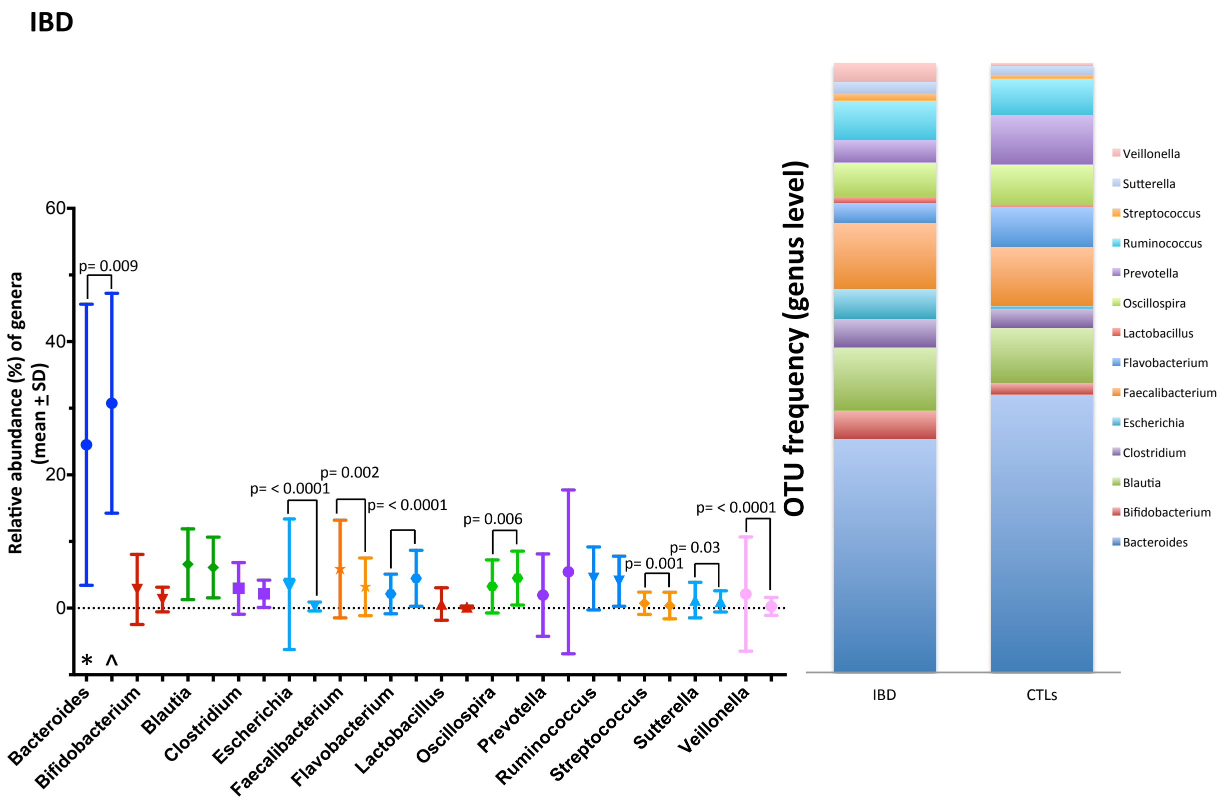

Figure S3 Microbiome taxonomic composition at genus level in IBD patients and control subjects (CTLs). Relative abundance of genera and OTU frequency are shown. Significant differences with p <0.05 are shown. *= patients; ^= controls.

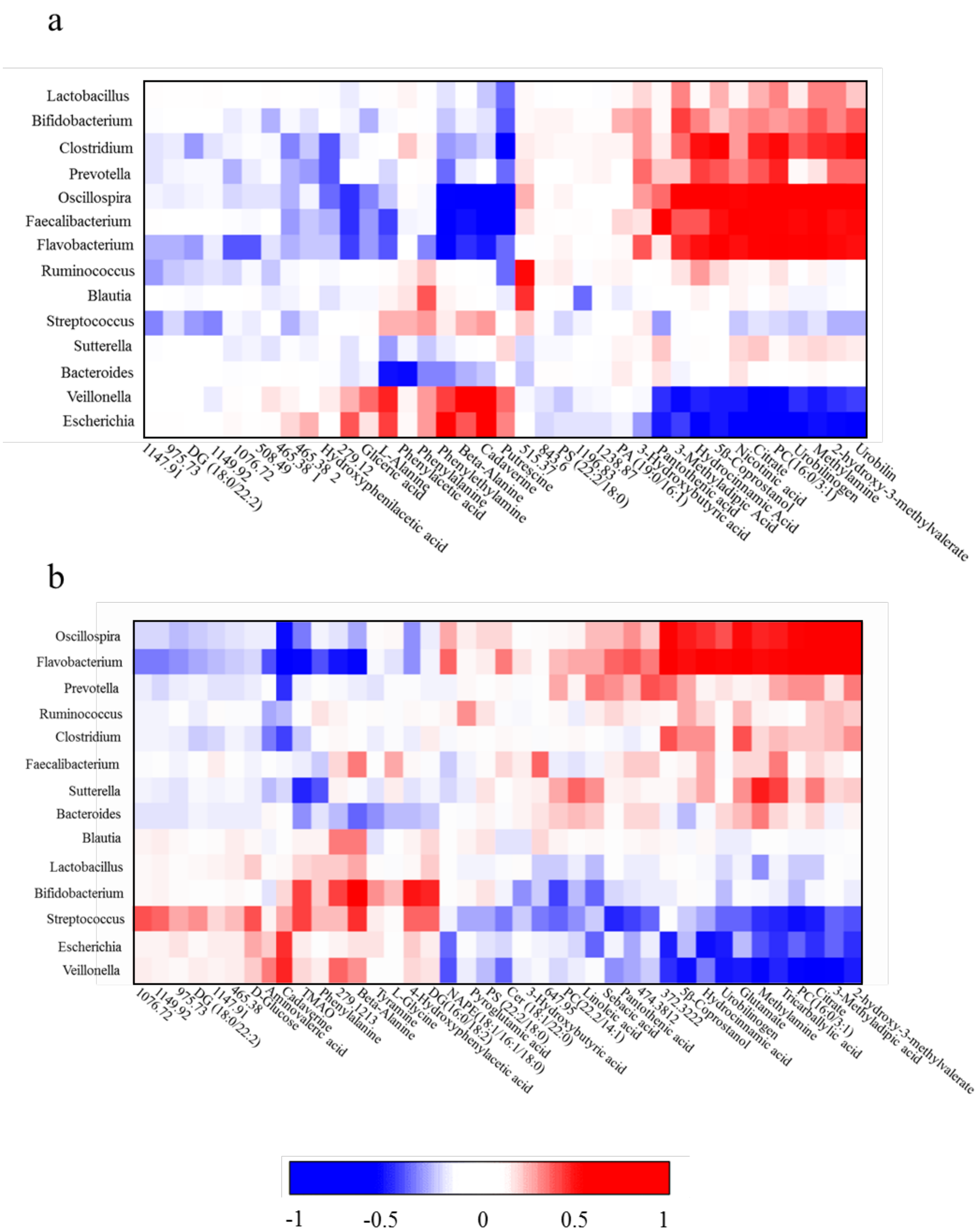

Figure S4 Inter-omic Spearman's rank correlation between metabolites and bacterial genera. Spearman correlation between statistically different metabolites and bacterial genera was calculated both for CD (**a**) and UC (**b**). All calculated correlations are shown.

a

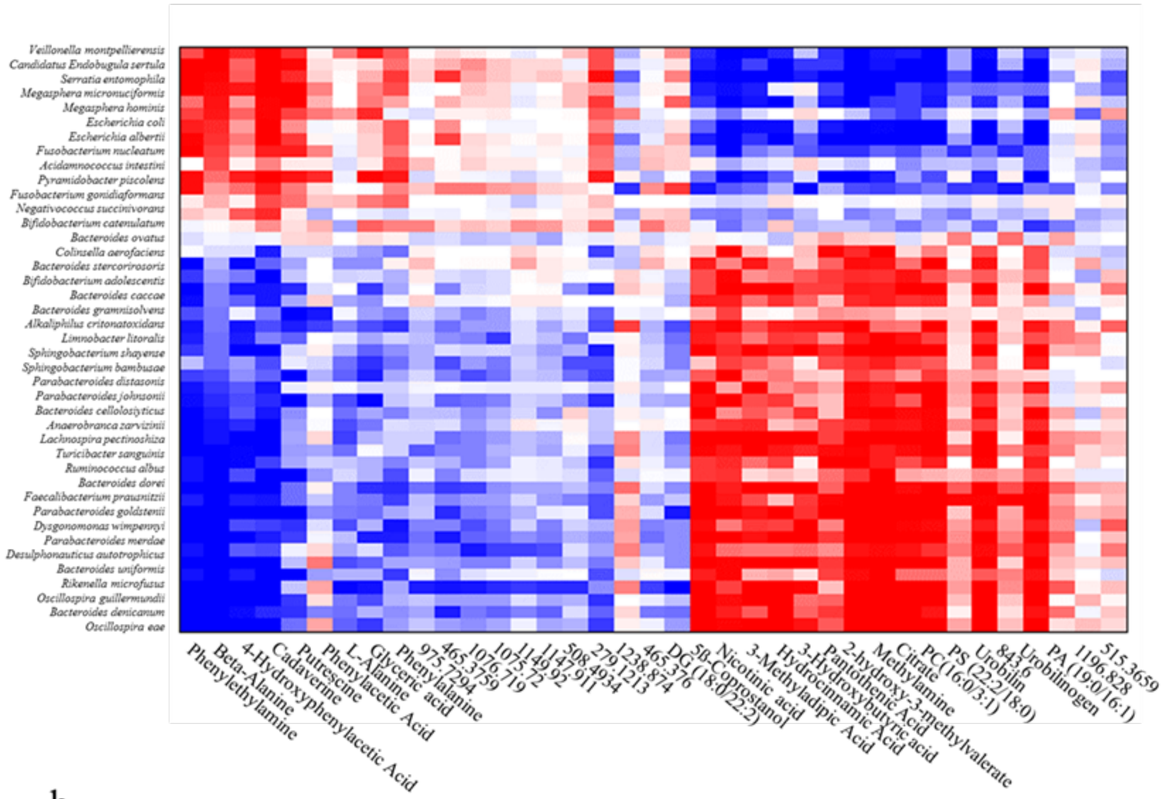

b

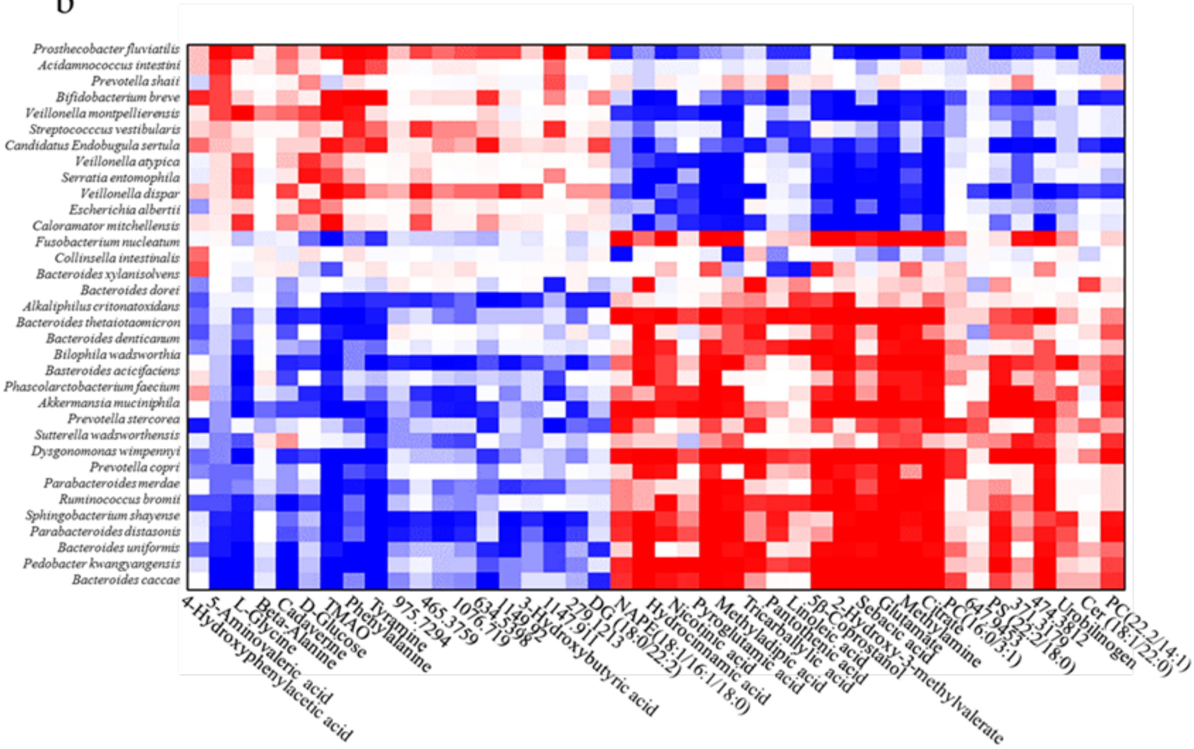

Figure S5 Inter-omic Spearman's rank correlation between metabolites and bacterial species. Spearman correlation between statistically different metabolites and bacterial species was calculated both for CD (**a**) and UC (**b**). All calculated correlations are shown.

References

1. Gregory KE, Bird SS, Gross VS, et al. Method development for fecal lipidomics profiling. Anal Chem 2012;85(2):1114-23.

2. R Core Team. R: A language and environment for statistical computing. R Foundation for Statistical Computing. 2011. http://www.R-project.org/.

3. Smith CA, Want EJ, O'Maille G, Abagyan R, et al. XCMS: processing mass spectrometry data for metabolite profiling using nonlinear peak alignment, matching, and identification. Anal Chem 2006;78:779-87.

4. Libiseller G, Dvorzak M, Kleb U, et al. IPO: a tool for automated optimization of XCMS parameters. BMC bioinformatics 2015;16:118.

5. Kuhl C, Tautenhahn R, Böttcher C, et al. CAMERA: an integrated strategy for compound spectra extraction and annotation of liquid chromatography/mass spectrometry data sets. Anal Chem 2012; 84:283-89.

6. Veselkov KA, Vingara LK, Masson P, et al. Optimized preprocessing of ultra-performance liquid chromatography/mass spectrometry urinary metabolic profiles for improved information recovery, Anal Chem 2011;83:5864-72.
